# Supplementary material for: Ehlers-Danlos: A Literature Review and Case Report in a Colombian Woman with Multiple Comorbidities
Source: Genes (Basel). 2022 Nov 15;13(11):2118. doi: 10.3390/genes13112118 (PMC9689997; doi:10.3390/genes13112118)
Supplement: Supplementary file 1 [file genes-13-02118-s001.zip › genes-1939691-supplementary.pdf]

	ullrich muscular dystrophy	bethlem myopathy	Myopathic Ehlers Danlos syndrome	Patient
Muscle weakness	yes	-	yes	yes
Proximal joint retractions	yes	yes	yes	yes
Hyperflexity of distal joints	yes	-	yes	yes
Slow progression of the disease	yes	yes	-	-
kyphosis	yes	-	escoliosis	yes
Skin abnormalities (follicular hyperkeratosis, keloid formation, velvet skin)	yes	yes	Yes, but other type of EDS	Yes, keloid formation
Ogival palate	yes	-	rare	yes
Difficulty for raising arms, exercising, climbing stairs	-	yes	-	
Hyperflexity of skin	-	-	yes	yes
Permanent muscle tension (contractures)	-	-	yes	yes
Delay in motor development	-	-	yes	-

Supplementary Table S1. Differential diagnosis of congenital myopathies. (-) data not available.

Chr	Ref	Alt	Gene	dbSNP	1000G ALL	1000G AMR	ExAC Freq	ExAC European (non-Finnish)	ESP 6500si ALL	gnomAD exome ALL	gnomAD exome European (non-Finnish)	III:7
6	C	T	COL12A1	rs201988277	0.00019968	0.001	0.00029	0.000431	0,0003	0.0003856	0.0004317	CT

Supplementary Table S2. Allelic frequencies of candidate variants under the prioritization criteria identified with the ANNOVAR tool. **Chr:** Chromosome. **Ref:** Reference allele. **Alt:** Alternate allele. **Gene:** Gene name. **dbSNP:** Variant identifier in dbSNP database. **1000GALL:** Allele frequency in 1000 genomes data base (all populations). **1000GAMR:** Allele frequency in 1000 genomes data base (Amerindian population). **ExACFreq:** Allele frequency in ExAC 65000 data base (all populations). **ExACAMR:** Allele frequency in ExAC 65000 data base (Amerindian population). **ESP6500siALL:** Allele frequency in NCBI-ESP 6500 database (all populations). **gnomADexomeALL:** Allele frequency in gnomAD database, exome data (all populations). **gnomADexomeAMR:** Allele frequency in gnomAD database, exome data (Amerindian population). **gnomADgenomeALL:** Allele frequency in gnomAD database, genome data (all populations). **gnomADgenomeAMR:** Allele frequency in gnomAD database, genome data (Amerindian population).

Chr	Ref	Alt	Gene	dbSNP	Geno Canyon	fitCons	GERP++RS	phyloP vertebrate	phyloP mammalian	phastCons vertebrate	phastCons mammalian	SiPhy	III:7
6	C	T	COL12A1	rs201988277	1	0,706	5,84	6,217	0,951	1	0,969	11,11	CT

Supplementary Table S3. Results of evolutionary conservation predictors of candidate variants under the prioritization criteria identified with the ANNOVAR tool. **Chr:** Chromosome. **Ref:** Reference allele. **Alt:** Alternate allele. **Gene:** Gene name. **dbSNP:** Variant identifier in dbSNP database. Evolutionary conservation predictors scores. **GenoCanyon:** Conservation scores with GenoCanyons tool (Conserved region=scores~1). **fitCons:** Conservation scores with fitCons tool: (Conserved region= ~1). **GERP++RS:** Conservation scores with GERP++RS tool (Conservation region=scores>4.4) **phyloPvertebrate:** Conservation scores with phyloP100 tool for vertebrates (Conservation region=scores>1.6). **phyloPmammalian:** Conservation scores with phyloP100 tool for mammalian (Conservation region=scores>1.6). **phastConsvertebrate:** Conservation scores with phastCons tool for vertebrates (Conservation region= scores~1). **phastConsmammalian:** Conservation scores with phastCons tool for vertebrates (Conservation region= scores~1) **SiPhy:** Conservation scores with SiPhy tool (Conservation region=scores >12.17).

Chr	Gene	dbSNP	SIFT	Poly phen2 HDIV	Poly phen2 HVAR	LRT	Mutation Taster	Mutation Assessor	FATHMM	PROVEAN	VEST3	Meta SVM	Meta LR	M-CAP	CADD	DANN score	Fathmm MKL	III:7
6	COL12A1	rs201988277	D	D	P	D	D	M	T	D	0,263	T	T	T	25,2	0,999	D	CT

Supplementary Table S4. Pathogenicity predictors results of candidate variants under the prioritization criteria identified with the ANNOVAR tool. **Chr:** Chromosome. **Gene:** Gene name. **dbSNP:** Variant identifier in dbSNP database. Pathogenicity predictors scores. **SIFT:** Pathogenicity prediction with SIFT tool: D=Deleterious, T=Tolerated). **Polyphen2HDIV:** Pathogenicity prediction with PolyPhem2 tool for Mendelian disease variants (D=Damaging, P=Possibly Damaging, B=Benign, U=Unknown). **Polyphen2HVAR:** Pathogenicity prediction with PolyPhem2 tool for all human disease-causing mutations (D=Damaging, P=Possibly Damaging, B=Benign, U= Unknown). **LRT:** Pathogenicity prediction with LTR tool (D=Deleterious, N=No Deleterious). **MutationTaster:** Pathogenicity prediction with Mutation Tester tool (A=Disease causing automatic, D=Disease causing, N=Polymorphism, P= Polymorphism automatic). **MutationAssessor:** Pathogenicity prediction with Mutation Assessor tool (N= Neutral effect, L=Low effect, M=Medium effect, H=High effect). **FATHMM:** Pathogenicity prediction with FATHMMM tool (D=Deleterious, T=Tolerated). **PROVEAN:** Pathogenicity prediction with PROVEAN tool (D=Deleterious, N=No Deleterious). **VEST3:** Pathogenicity SCORES with VEST tool (Deleterious=escores>0.63). **MetaSVM:** Pathogenicity prediction with MetaSVM tool (D=Damaging, T=Tolerated). **MetaLR:** Pathogenicity prediction with MetaLR tool (D=Damaging, T=Tolerated). **M-CAP:** Pathogenicity prediction with M-CAP tool (D=Damaging, B=Benign). **CADD:** Pathogenicity scores with CADD tool (Deleterious=scores>14). **DANN:** Pathogenicity prediction with DANN tool (Pathogenic= scores~1). **FathmmMKL:** Pathogenicity prediction with FathmmMKL tool (D=Deleterious, T=Tolerated).

Chr	Gene	Change	dbSNP	221 F1III:7	Varsome	Intervar	Clinvar
6	COL12A1	p.Thr2618Met	rs201988277	CT	PM2, PP2, PP3 (uncertain significance)	PM1, PM2, PP5 and BS2 (uncertain significance)	Uncertain significance

Supplementary Table S5. Clinical interpretation of the candidate variants identified in the exome analysis in a proband with congenital myopathy. **Chr:** Chromosome. **Gene**: Name of the gene. **Change:** Nucleotide/amino acid change. **dbSNP:** Identifier of the variant in the dbSNP database. **Varsome:** Classification of variants according to the Varsome platform. **Intervar:** Classification of the variants according to the Intervar platform. **PM1:** Located in a mutational hot spot and/or critical and well-established functional domain (e.g., active site of an enzyme) without benign variation**. PM2:** Absent from controls (or at extremely low frequency if recessive) in Exome Sequencing Project, 1000 Genomes Project, or Exome Aggregation Consortium. **PP2:** Missense variant in a gene that has a low rate of benign missense variation and in which missense variants are a common mechanism of disease. **PP3:** Multiple lines of computational evidence support a deleterious effect on the gene or gene product (conservation, evolutionary, splicing impact, etc.). **PP5:** Reputable source recently reports variant as pathogenic, but the evidence is not available to the laboratory to perform an independent evaluation. **BS2:** Observed in a healthy adult individual for a recessive (homozygous), dominant (heterozygous), or X-linked (hemizygous) disorder, with full penetrance expected at an early age.
